# High blood pressure and aeronautical fitness: experience at the aeromedical expertise center of Rabat

**DOI:** 10.11604/pamj.2024.47.41.42262

**Published:** 2024-02-01

**Authors:** Fahd Bennani Smires, Zakaria Iloughmane, Mouna Elghazi, Meryem Zerrik, Houda Echchachoui, Mohamed Chemsi

**Affiliations:** 1 Aviation Medicine, Aeromedical Expertise Center of Rabat, Hôpital Militaire d´Instruction Mohamed V, Rabat, Maroc

**Keywords:** High blood pressure, aeronautical fitness, aircrew

## Abstract

High blood pressure is a major cardiovascular risk factor closely linked to serious cardiovascular events. A real public health problem affecting more than one in three adults. Aircrew does not escape this pathology, despite very strict medical selection and rigorous and regular medical monitoring by the aircrew doctor during revision visits. We conducted a retrospective study at the medical expertise center for aircrew in Rabat which made it possible to collect 34 hypertensive civilian aircrew for 10 years, from January 2012 to December 2022. The median age at the time of the study was 56.5. The aeronautical specialties practiced by our aircrew population were dominated by class 1. The prevalence of hypertension in Moroccan civilian aircrew: out of 2000 monitored annually at the aeromedical expertise center for 10 years, 34 cases were collected, i.e.: 1.7%. The average age of discovery was 49 years and in 23 cases the diagnosis was established by systematic screening during periodic fitness visits. More than 24 aircrews had no family history of hypertension. On the therapeutic level, lifestyle and dietary measures were systematically prescribed in all our aircrew, 18 patients were put on monotherapy, 11 on dual therapy, and 2 on triple therapy. Compared to fitness decisions, they were variable according to the grade of hypertension, the control of complications, and the aeronautical function. The discovery of hypertension in aircrew can jeopardize aviation safety with the risk of subtle or sudden incapacity in flight through neurological or cardiovascular complications, which could impact the fitness decision. However, advances in medicine and the management of hypertension made in recent years have prompted the medical and aeronautical authorities to revise the standards of aptitude.

## Brief

High blood pressure (HBP) is a major cardiovascular risk factor linked to serious cardiovascular events such as myocardial infarction, heart failure, or stroke. Its prevalence continues to increase due to the aging of the population and changing lifestyles [1]. A real public health problem affecting more than one in three adults, hypertension is responsible for 9.4 million deaths each year. in the USA, it is estimated that high blood pressure represents 8.3% of cardiovascular causes of death [2]. The aircrew population is not spared; we understand why this pathology is so feared in aviation medicine, despite a very strict medical selection and rigorous and regular medical monitoring by aeronautical doctors during revision visits. The objective of this work, conducted at the Aircrew Medical Expertise Center (AMEC) of the Mohammed V Military Instruction Hospital in Rabat, concerning high blood pressure among civilian aircrew for 10 years (2012-2022 ) is to study the prevalence and particularities of hypertension among Moroccan civilian aircrew, to evaluate the management of hypertensive civilian aircrew, to explain the expert´s approach in HBP cases and study the resulting decisions ensuing, to demonstrate the preventive and predictive role of the aeronautical medical expert, to preserve flight safety, and finally, to report the experience of the aircrew medical expertise center.

We retrospectively analyzed 34 files of civilian aircrew, monitored for HBP and who met our inclusion criteria, the study was carried out from January 2012 until December 2022 (10 years). Inclusion criteria were male and female flight crews with HBP during their career and who continue to be regularly monitored at the center were included. Excluded from the study were military flight crews, flight crews lost to follow-up, flight crews with incomplete files, and hypertensive patients discovered during the admission visit. The main limitation of the study was the small size of our sample of flight personnel (FP) suffering from hypertension. Administrative data were medical data (heredity, associated cardiovascular risk factors), circumstances of discovery, and suitability decisions.

Our sample includes 34 flight personnel monitored at the aircrew medical expertise center of the Mohammed V Military Instruction Hospital in Rabat, including 24 males and 10 females; i.e. a M/F sex ratio of 2: 4. The average age of our flight personnel at the time of the study was 56.5 years with extremes ranging from 39-68 years. The aeronautical specialties exercised by our population were dominated by air traffic controllers. The absence of class 2 pilots is explained by the fact that they are followed by approved doctors outside the aircrew medical expertise center (Table 1).

**Table 1:** distribution of flight crews according to gender and aeronautical specialty

	Aeronautical specialties			
	Classe 1	Classe 2	Classe 3	Classe 4
Female	Captain: 0	Private pilot: 0	Air controller: 6	Steward: 0
	Air pilot officer: 0			Flight attendant: 4
Male	Captain: 9	Private pilot: 0	Air controller: 9	Steward: 3
	Air pilot officer: 3			Flight attendant: 0

**Figure 1:**
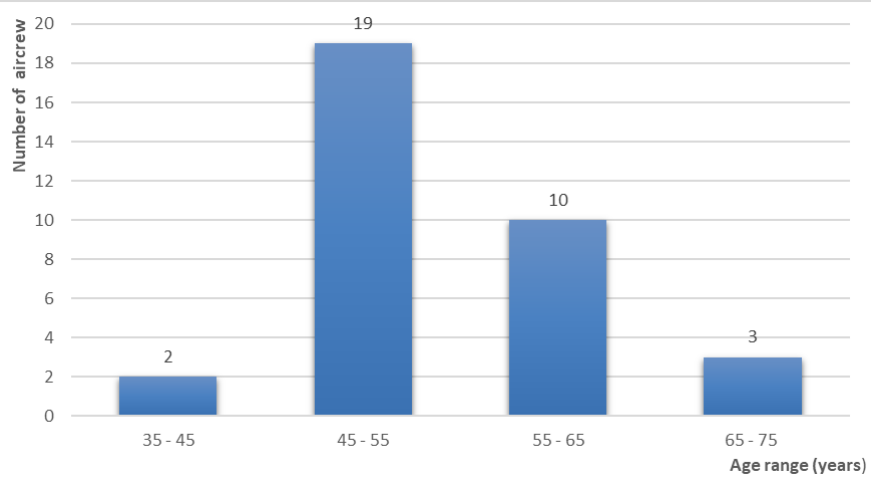
distribution of flight personnel according to age of discovery of high blood pressure

**Figure 2:**
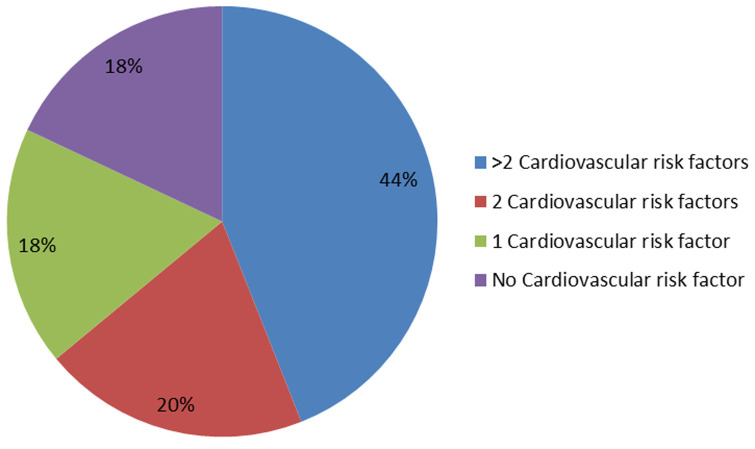
accumulation of cardiovascular risks associated with high blood pressure outside of age and male sex

**Prevalence among Moroccan aircrew:** out of 2000 personnel followed annually at the AMEC for 10 years, there were 34 cases: 1.7%. The average age of discovery was 49 years with a minimum of 37 years and a maximum of 55 years (Figure 1). The heredity of HBP was present in 10 FP, in a first-degree relative, while 24 patients had no family history of arterial hypertension. The associated cardiovascular risk factors most encountered in our sample of FP studied are essentially represented by: age > 50 years, heredity, male sex, dyslipidemia, smoking, and diabetes (Figure 2).

Hygiene-dietary measures were systematically prescribed to all our flight personnel; 31 cases required the additional use of antihypertensive medications. In our sample, suitability decisions varied: 3 cases under hygiene-dietary measures alone require an annual check-up, and for the 18 cases under monotherapy, monitoring is done every six months. When the aptitude standards are exceeded, 13 cases of dual therapy require quarterly monitoring with an exemption. One flight personnel was declared unfit for severe hypertension on dual therapy.

In aviation medicine, the flight crew does not escape this pathology, despite very strict medical selection (elimination of hypertension on admission) and rigorous regular medical monitoring by the aeronautical doctors during revision visits (screening and correction of cardiovascular risk factors). This is a young population with a relatively high socio-cultural level and exercising a profession with sometimes operational and stressful missions requiring high physical and mental performance, especially in a military environment and in degraded flight conditions. Several factors are involved in the etiopathogenesis of hypertension in aircrew, in addition to the lifestyle (stress, sedentary lifestyle, excess weight) generated by nature and the workload, especially among transport pilots, dietary constraints during missions, long-haul flights and night flights, are added aeronautical specific factors in particular, jet lag in a civilian environment and acceleration in the military environment [3]. The discovery of hypertension in flight crew can jeopardize aviation safety with the risk of subtle or sudden incapacity in flight through its neurological or cardiovascular complications, notably coronary heart disease and strokes, as it can act of hypotensive phenomena by dysautonomia neurovegetative which can cause cardiovascular adaptation disorders to linear accelerations as well as an alteration of vision due to ophthalmological damage [4].

The management of HBP in aircrew does not fundamentally differ from its management in the general population; it must go through several stages: the characterization of hypertension, the etiological investigation, the study of the impact, and finally the therapeutic decision. Although high blood pressure is not a frequent reason for hospitalization, it gives rise to the greatest number of chronic drug treatments among aircrew [5], in our population, one in two subjects receives medication.

The progress in medicine has pushed the medical-aeronautical authorities to revise the fitness standards. Essential hypertension can remain compatible with aeronautical fitness, if it is mild to moderate, without significant visceral impact, with effective antihypertensive treatment, without side effects, and compatible with aviation safety [6]. In fact, out of 34 hypertensive flight personnel, only one pilot was declared permanently unfit, the other 33 cases were able to be rehabilitated by derogation from medical standards. High blood pressure represents a situation of high cardiovascular risk. Its definition has varied over the years; it is also recognized as arbitrary but necessary to establish recommendations. It constitutes a threat to aviation safety. This threat can be expressed immediately through vascular damage (stroke), or it will be integrated into the general framework of cardiovascular risk factors increasing the risk of a major cardiovascular event (acute coronary syndrome).

In hypertensive PN, complications are capable of jeopardizing the safety of flight due to the risk of subtle or sudden incapacity in flight that may occur during a complication This study therefore allowed us to confirm that the management of hypertension in civilian FP must be adapted to their specific conditions. Screening for hypertension must be carried out before the onset of initial complications, which is done for the majority of patients in our study. Treatment of hypertension is of paramount importance in preventing damage cerebrovascular and long-term cardiovascular complications. The fight against cardiovascular risk factors must remain a priority for expert doctors since aeronautical medicine is responsible for the regular monitoring of FP without forgetting its role in health education. The appearance of hypertension in an aircrew is not synonymous with unfitness to fly, rehabilitation is possible under certain conditions. However, these should not call into question flight safety, which remains the constant obsession of the expert.
